# Sestrin2, a Regulator of Thermogenesis and Mitohormesis in Brown Adipose Tissue

**DOI:** 10.3389/fendo.2015.00114

**Published:** 2015-07-24

**Authors:** Seung-Hyun Ro, Ian Semple, Allison Ho, Hwan-Woo Park, Jun Hee Lee

**Affiliations:** ^1^Department of Molecular and Integrative Physiology, University of Michigan, Ann Arbor, MI, USA; ^2^Department of Cell Biology, College of Medicine, Konyang University, Daejeon, South Korea

**Keywords:** Sestrin2, brown adipose tissue, mitochondria metabolism, thermogenesis, reactive oxygen species, antioxidants, mitohormesis, aging

## Abstract

Sestrin2 is a stress-inducible protein that functions as an antioxidant and inhibitor of mTOR complex 1. In a recent study, we found that Sestrin2 overexpression in brown adipocytes interfered with normal metabolism by reducing mitochondrial respiration through the suppression of uncoupling protein 1 (UCP1) expression. The metabolic effects of Sestrin2 in brown adipocytes were dependent on its antioxidant activity, and chemical antioxidants produced similar effects in inhibiting UCP1-dependent thermogenesis. These observations suggest that low levels of reactive oxygen species (ROS) in brown adipocytes can actually be beneficial and necessary for proper metabolic homeostasis. In addition, considering that Sestrins are ROS inducible and perform ROS detoxifying as well as other metabolism-controlling functions, they are potential regulators of mitohormesis. This is a concept in which overall beneficial effects result from low-level oxidative stress stimuli, such as the ones induced by caloric restriction or physical exercise. In this perspective, we incorporate our recent insight obtained from the Sestrin2 study toward a better understanding of the relationship between ROS, Sestrin2, and mitochondrial metabolism in the context of brown adipocyte physiology.

## Introduction: Sestrins

Sestrins are a group of stress-inducible proteins, which are highly conserved across species and have two distinct biologically active functions (1). First, they function as an antioxidant that suppresses reactive oxygen species (ROS) accumulation (2, 3) through a poorly characterized biochemical mechanism (4), which may involve regulation of antioxidant transcription factors (5). Independently of this redox function, Sestrins also act as feedback inhibitors of mechanistic target of rapamycin complex 1 (mTORC1) through activation of AMP-activated protein kinase (AMPK) (6, 7) or through inhibition of Rag GTPases (8–11). Through these functions, Sestrins have been shown to attenuate multiple age- and obesity-associated metabolic pathologies, such as fat accumulation, glucose intolerance, insulin resistance, mitochondrial dysfunction, muscle degeneration, and cardiac malfunction (5–7, 12–15). Because the expression of Sestrins is induced upon a variety of environmental stresses, such as DNA damage, oxidative stress, and hypoxia (2), Sestrins are considered a mechanistic link between stress and aging (16).

## ROS as Signaling Molecules

Reactive oxygen species are a group of free oxygen radicals and reactive non-radicals, such as superoxide $\left(O_{2}^{\cdot -}\right)$, hydroxyl radical (OH^⋅^), nitric oxide (NO^⋅^), and hydrogen peroxide (H_2_O_2_) (17–19). In mammalian cells, ROS can be generated from various sources, such as mitochondria, peroxisomes, and ROS-producing cytosolic enzymes, such as NADPH oxidases (20–23). Under normal physiological conditions, the intracellular levels of ROS are homeostatically controlled. However, aberrantly increased ROS levels can damage intracellular organelles and critical macromolecules, such as proteins, DNA, and lipids. The ROS-induced oxidative damage can contribute to the development of numerous pathological disorders, such as cardiovascular disease, neurodegenerative diseases, mitochondrial disease, obesity, diabetes, cancer, and aging (24–30).

Although ROS were initially considered to be undesired byproducts of metabolism (31), a modernized view of ROS has emerged; ROS are now considered to be important signaling molecules (32–35). ROS can control diverse signaling pathways, including MAPK/ERK1/2 pathway (36, 37), PI3K/Akt pathway (38, 39), IKK/NF-kB pathway (40–42), and p38 MAPK pathway (43, 44), which are critically involved in cell growth, differentiation, metabolism, and inflammation. Through these pathways, ROS contribute to the maintenance of physiological homeostasis in cells and tissues (45–49). These new findings support the concept of mitohormesis, which explains how physiological ROS can be beneficial to the cells and organism; ROS serve as sub-lethal stressors that act as signaling molecules to induce endogenous defense mechanisms, which ultimately improve mitochondrial metabolism and promote stress resistance, metabolic health, and longevity (50–52).

## Role of ROS in BAT Metabolism

BAT is the organ mainly responsible for non-shivering thermogenesis, which is mediated by uncoupling protein 1 (UCP1) (53–55). UCP1 is the key protein for thermogenesis, which is specifically induced in BAT upon exposure to cold temperature. Low temperatures activate p38 MAPK by stimulating sympathetic neurons and inducing cAMP accumulation in BAT (56–58). Activated p38 MAPK subsequently activates several transcription factors, such as ATF-2 and PGC-1α, which induces UCP1 expression in BAT (58, 59). By translocating into the mitochondrial inner membrane and dissipating the proton gradient, UCP1 uncouples mitochondrial respiration from ATP synthesis and generates heat (53, 60). UCP1-dependent thermogenesis in BAT increases energy expenditure reduces body fat and improves metabolic homeostasis (60, 61).

Chronic accumulation of ROS produced by dysfunctional mitochondria may deteriorate the BAT metabolism (62, 63), which can be one of the mechanisms of how aging and obesity interferes with BAT metabolism (64–66). In this respect, suppressing excessive ROS might be key to reinforcing BAT metabolism against aging. Chemical antioxidants, artificial or naturally occurring substances, serve to scavenge ROS, many of which are byproducts of cellular metabolism, which when in excess can cause oxidative damage and promote disease development and aging (26, 32). Most cells utilize various antioxidant enzymes, such as catalases, superoxide dismutases, glutathione peroxidases, peroxiredoxins, and Sestrins, which serve to reduce the negative consequences of ROS accumulation (3, 33, 67). We have recently investigated the role of ROS in BAT metabolism through the use of chemical antioxidants, such as butylated hydroxyanisole (BHA) and *N*-acetylcysteine (NAC), as well as forced overexpression of Sestrin2 (68).

Surprisingly, our recent results indicated that physiological levels of ROS could also play a critical role in thermogenic processes (68). BHA and NAC, as well as Sestrin2 overexpression, resulted in a strong reduction of UCP1 expression in BAT. Subsequent *in vitro* and *in vivo* studies showed that physiological ROS in BAT potentiate cAMP-induced p38 MAPK activation, which mediates cold-induced UCP1 expression. Correspondingly, Sestrin2-overexpressing mice were unable to upregulate UCP1 or generate heat in response to cold exposure. These results demonstrate that ROS are critical for proper BAT metabolism. Therefore, prolonged antioxidant treatment may interfere with thermogenesis and possibly other ROS-dependent physiological processes. This idea is consistent with recent reports where administered antioxidants had neutral or negative effects on health and life span in animals and humans (69, 70).

## Caloric Restriction and Physical Exercise as Potential Inducers of ROS and Mitohormesis

Caloric restriction, being defined as a reduction in *ad libitum* calorie uptake, has been shown to extend life span in a variety of organisms (71). Notably, emerging evidence indicates that caloric restriction is capable of increasing mitochondrial metabolism, such as oxidative respiration, in yeast (72), *Caenorhabditis elegans* (73) and *Drosophila* (74, 75). Increased production of ROS as a consequence of increased mitochondrial respiration has been suggested to be a critical regulator of life span during caloric restriction (73, 76–79). Caloric restriction-induced ROS can stimulate antioxidant defense mechanisms, such as radical-scavenging enzymes, that mediate various mitohormetic responses (5, 73, 80–82). Carbohydrate-deficient diets also increase oxidative metabolism, subsequently resulting in enhanced ROS defense (83). Therefore, these recent studies suggest that caloric restriction increases longevity at least partially by inducing oxidative metabolism and a mitohormetic defense system.

Physical exercise undoubtedly provides positive effects on diverse diseases, such as obesity, type 2 diabetes, cardiovascular disease, cancer, and general aging (84–88). Similar to caloric restriction, exercise is capable of increasing mitochondrial biogenesis, oxidative metabolism, and mitochondrial ROS production (89–92). Many studies propose that exercise-induced ROS contributes to mitohormesis, which increases health span and mean life span (93–97). Because co-treatment with antioxidants prevented the beneficial mitohormetic response of physical exercise (85, 98), physiological ROS produced by exercise was considered critical for the benefits of exercise. Therefore, both caloric restriction and exercise seem to utilize physiological ROS induction as a means to induce antioxidant defense and promote the life and health span of an organism.

## Sestrins as Potential Regulators of Mitohormesis

Sestrins have been identified as important regulators of age- and obesity-associated pathologies in diverse tissues including liver, adipose, and muscle (5–7, 12–15, 68). Sestrins are thought to attenuate tissue aging through their dual biological activities in reducing ROS and inhibiting mTORC1 (1, 2, 16). Excessive accumulation of ROS and chronic activation of mTORC1 signaling are well-known promoters of tissue aging. Considering that Sestrins are transcriptionally activated upon oxidative stress, it is highly likely that Sestrins may be regulators of mitohormesis. For example, upon a non-toxic level of ROS stimuli, Sestrins may be induced to perform antioxidant and mTORC1-suppressive functions to defend against oxidative damage and attenuate tissue aging.

It should also be noted that, although critical for attenuating tissue aging, the loss of Sestrins *per se* does not substantially reduce life span of *Drosophila* (1) or *C. elegans* (15). This could be because there are unknown compensatory mechanisms instigated by the loss of Sestrins, which are capable of maintaining life span, but are not sufficient to restore health span. However, Sestrin-deficient *C. elegans* is hypersensitive to oxidative stress (15), suggesting that Sestrin is indeed critical for the stress adaptation of an organism. It has been shown in the same organism that low levels of oxidative stress (conferred by a chemical that induces mitochondrial ROS) can increase the life span of *C. elegans* (99, 100). Caloric restriction in *C. elegans* extends life span partially by inducing mitochondrial production of ROS (73). Therefore, it would be very interesting to investigate if Sestrins are indeed regulators of the mitohormetic effect in these aging models.

## Role of Sestrin2 in BAT Metabolism

Although the beneficial role of Sestrins in attenuating tissue aging and obesity-associated metabolic pathologies has been clear in several tissues, such as liver and skeletal/cardiac muscle (6, 7, 12, 13), the role of Sestrin2 in BAT metabolism seems to be more complicated. As discussed above, Sestrin2 overexpression in BAT interferes with proper UCP1 expression and mitochondrial uncoupling (68). Even though Sestrin2 overexpression activated AMPK and subsequently promoted mitochondrial biogenesis, these beneficial effects were nullified by the drastic effect of UCP1 loss. As a result, the Sestrin2-overexpressing mice were defective in BAT thermogenesis and exhibited increased fat accumulation (68).

Interestingly, loss of Sestrin2 also interfered with proper BAT metabolism. Although UCP1 expression was relatively increased in BAT of Sestrin2-deficient mice, the whitening of BAT due to fat accumulation is markedly increased upon Sestrin2 deficiency (68). Analysis of mRNA markers for mitochondrial biogenesis and quantification of mitochondrial DNA suggests that BAT from Sestrin2-deficient mice exhibited decreased mitochondrial contents (68). This could be because Sestrin2 plays a critical role in producing sufficient amounts of mitochondria in BAT, which would be necessary for proper energy dissipation and homeostatic BAT metabolism. Sestrin2 may promote mitochondrial biogenesis through activation of AMPK (7, 12, 101, 102) and subsequent upregulation of PGC-1α activity (103, 104). Therefore, although overexpression of Sestrin2 can interfere with the physiological level of ROS necessary for thermogenesis control, endogenous Sestrin2 still plays a critical role in maintaining mitochondrial homeostasis.

## Conclusion

**Figure 1:**
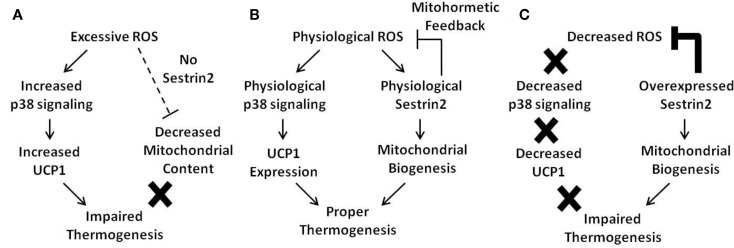
**Current model of the mitohormetic relationship between ROS, Sestrin2, and mitochondrial thermogenesis**. Under conditions of excessive or decreased ROS, BAT thermogenesis is impaired. However, a physiological level of ROS is able to maintain proper BAT metabolism. **(A)** In cases where antioxidant activity is low, such as in the absence of Sestrin2, ROS levels are upregulated. Excessive ROS may directly impair mitochondrial homeostasis by direct damage to the organelle. In addition, Sestrin2 is critical for mitochondrial biogenesis. Therefore, in the absence of Sestrin2, BAT mitochondrial content is decreased as well as its thermogenic capacity. **(B)** When Sestrin2 is physiologically expressed, an adequate level of ROS is produced to ensure proper p38 MAPK activation and UCP1 expression. Physiological Sestrin2 expression promotes mitochondrial biogenesis, and the mitohormetic mechanism conferred by Sestrin2 is necessary to maintain proper BAT metabolism such as thermogenesis. **(C)** When Sestrin2 is overexpressed, ROS are dramatically suppressed. Because ROS are critical for UCP1 expression in BAT, Sestrin2 overexpression decreases UCP1 expression. Although mitochondrial biogenesis is enhanced by Sestrin2 overexpression, decreased UCP1 expression prohibits proper thermogenesis in BAT.

Upon diverse environmental stresses including oxidative stress, Sestrin-family proteins are transcriptionally upregulated to reduce pathogenic levels of ROS and suppress chronic activation of mTORC1 signaling. As chronic ROS accumulation and prolonged mTORC1 activation are both detrimental for metabolic homeostasis, Sestrins are potential regulators of mitohormesis, which is a beneficial metabolic effect of low-level ROS production. Sestrins may also play a critical metabolism-controlling role in BAT. However, because ROS are also critical for UCP1 expression and subsequent mitochondrial uncoupling, artificial overexpression of Sestrin2 and subsequent elimination of ROS interfered with non-shivering thermogenesis, which is one of the most critical physiological functions of BAT. Therefore, it is highly likely that ROS levels, Sestrin2 expression, and mitochondrial metabolism are connected to each other through a complicated and finely coordinated network, and a delicate balance between these components seems to be critical for proper BAT homeostasis (Figure 1).

## Conflict of Interest Statement

The authors declare that the research was conducted in the absence of any commercial or financial relationships that could be construed as a potential conflict of interest.
